# On Beeswax

**Published:** 1868-03

**Authors:** Jas. F. Babcock


### ARTICLE VI.

### *On Beeswax.*

By Jas. F. Babcock.

The name *wax* is given to quite a number of bodies of very different origin, of which that secreted by the *Apis mellifica* is the type.

It is found in the pollen of most flowers, in the amenta of the birch, hazel, willow, oak, and in solution in the milky juice of the cow-tree. The brilliant surface of the petals of flowers is due to it. The surface of the stalk of the sugar-cane, the green fecula of the cabbage, the stones and skins of many fruits and the berries of the *Myrica angustifolia*, *latifolia*, as well as the *cerifera*, afford it in greater or less proportion.

A fertile specimen of the latter will yield about seven pounds of berries, which contain twenty-five per cent. of wax. The wax from plants is extracted by boiling them in water, to the surface of which the wax rises, and, on cooling, may be easily removed.

It is not proposed to describe these varieties of wax, but to confine the essay strictly to the title. The following table gives the principle properties of these different bodies, as well as that of ordinary beeswax, both bleached and yellow.

| | C | H | O | Melt'g point. | spec. gr. | |
|---|---|---|---|---|---|---|
| Beeswax, yellow | 80.20 | 13.44 | 6 36 | 149°F. | | Lewy. |
| " bleached | 79 20 | 13.15 | 7.65 | 157°F. | .996 | Lewy. |
| Vegetable wax, Japan | 70 00 | 12 07 | 17.93 | 104°F. | .970 | Thompson. |
| Myrtle wax | 74 23 | 12.07 | 13.70 | 109°F. | 1 015 | Giradin. |
| Brazil wax | 71 88 | 12.03 | 16.09 | 206°F. | .980 | Brande. |
| Cow-tree wax | | | | 137°F. | .969 | Thompson. |
| Palm wax | 80.28 | 13.20 | 6.52 | 161°F. | | Lewy. |

The wax of vegetables, with the exception of Japan wax, is less combustible, and less easy to bleach than that produced by certain insects of the order *Hymenoptera*, particularly the honey-bee.

These insects secrete the wax under the rings of their abdomen, and construct with it the hexagonal cells into which they deposit their eggs and honey. To procure the wax, the honey-comb is pressed to separate the honey from the wax. The cakes thus formed are thrown into boiling water, which dissolves what honey still adheres to the wax, which melts, and, rising to the surface, forms, on cooling a solid cake. This being separated and remelted, forms the crude yellow wax of commerce.

In this state wax owes its color, its aromatic odor, and its peculiar consistence, to foreign bodies, and, in part, to a small amount of honey.

It is bleached by the French in the following manner: It is melted in copper vessels, and after complete liquefaction, is agitated with 8 oz. of pulverized cream of tartar for each 100 lbs. After some minutes' agitation it is allowed to deposit its impurities, and is drawn into a wooden vessel and allowed to deposit a further amount of foreign substance,—dirt, sand, bees, etc.—and while still liquid, is drawn upon a little roller partly immersed in water, to which a regular rotation is given,—thus producing thin sheets or ribbons of wax, which may be detached from the roller, being now ready for the process of bleaching. This is accomplished by the exposure of the yellow scales and ribbons, upon cloths, to the direct rays of the sun and the dew, for several days, during which time the wax completely loses its color. It is, however, in practice impossible to bleach the wax at a single operation, as the effect takes place only on the surface, and, as the ribbons have a certain thickness, it is necessary to melt them anew, and having repeated the operation of granulating, it is submitted to a second exposure. The wax thus bleached is melted, and cast into discs of one or two ounces weight, and forms the *Cera alba* of the Pharmacopœia.

Wax from different localities does not bleach with equal facility. That from the East, from Barbary and from the central portions of France, is bleached with ease. Wax from Brazil is bleached with much difficulty.

Chlorine cannot be used to bleach wax—at least when the wax is to be used for medicinal purposes, or for candles.

It is bleached by this gas, but it combines with it, and liberates one equivalent of hydrogen.

It was in examining the action of chlorine upon wax that Gay-Lussac discovered the principles of substitution.

I am not aware of any experiments having been made with sulphurous acid, which it is not unlikely might prove of service in this direction.

Beeswax, when pure, has neither taste nor smell; as is seen by the table, it melts at 157° F. and is of a specific gravity of 966. It burns without smoke or disagreeable odor.

It does not furnish, by destructive distillation, either sebacic acid or acroleine, which property affords a very simple method for ascertaining the absence of tallow, fat, or any body containing stearine, oleine, or margarine, which, under the same circumstances, furnish more or less of these substances.

It is insoluble in water, but soluble in all proportions in the fixed and volatile oils, bisulphide of carbon, and benzine. Its complete solution in these substances demonstrates its freedom from fecula, sulphur, sawdust, or bone-dust, which have been found in the wax of commerce, sometimes amounting to 60 per cent. of the whole weight.

Several bodies have been isolated from beeswax: ceroleine, amounting to about four per cent.; myricine, thirty per cent.; and cerine, sixty-five per cent., being among the number.

It is saponified with greater difficulty than fatty bodies, but furnishes a handsome soap,—a product holding a prominent place among the chemical *novelties* in the British section of the Paris Exposition.

The abundance and low price of paraffine have made this substance one of the principal articles used in the falsification of wax, and perhaps of all others it is the least objectionable, being without marked physiological effect upon the system.

In answer to the last portion of the query,—wax substitutes—it appears to the writer that paraffine is capable of taking the place of wax to a much greater extent than has been supposed. When melted with oils, it forms crystaline scales on cooling; but this property is entirely destroyed by the addition of five to ten per cent. of wax,—this addition causing the mixture to cool in a homogeneous mass, without crystallization.—*Proceedings of Ph. Ass.*, 1867.—*Druggists' Circular.*

